# Transcranial DC stimulation modifies functional connectivity of large‐scale brain networks in abstinent methamphetamine users

**DOI:** 10.1002/brb3.922

**Published:** 2018-02-15

**Authors:** Alireza Shahbabaie, Mitra Ebrahimpoor, Ali Hariri, Michael A. Nitsche, Javad Hatami, Emad Fatemizadeh, Mohammad Ali Oghabian, Hamed Ekhtiari

**Affiliations:** ^1^ Institute for Cognitive Science Studies Tehran Iran; ^2^ Neuroimaging and Analysis Group Research Center for Cellular and Molecular Imaging Tehran University of Medical Sciences Tehran Iran; ^3^ Iranian National Center for Addiction Studies Tehran University of Medical Sciences Tehran Iran; ^4^ Department of Psychology and Neurosciences Leibniz Research Center for Working Environment and Human Factors Dortmund Germany; ^5^ Department of Medical Statistics and Bioinformatics Leiden University Medical Center Leiden the Netherlands; ^6^ Department of NanoEngineering and Materials Science and Engineering Program University of California San Diego La Jolla CA USA; ^7^ Department of Psychology and Educational Sciences University of Tehran Tehran Iran; ^8^ Department of Electrical Engineering Sharif University of Technology Tehran Iran

**Keywords:** functional connectivity, large‐scale brain networks, methamphetamine craving, noninvasive brain stimulation, resting state fMRI, transcranial direct current stimulation

## Abstract

**Background:**

Transcranial direct current stimulation (tDCS) is a noninvasive brain stimulation tool suited to alter cortical excitability and activity via the application of weak direct electrical currents. An increasing number of studies in the addiction literature suggests that tDCS modulates subjective self‐reported craving through stimulation of dorsolateral prefrontal cortex (DLPFC). The major goal of this study was to explore effects of bilateral DLPFC stimulation on resting state networks (RSNs) in association with drug craving modulation. We targeted three large‐scale RSNs; the default mode network (DMN), the executive control network (ECN), and the salience network (SN).

**Methods:**

Fifteen males were recruited after signing written informed consent. We conducted a double‐blinded sham‐controlled crossover study. Twenty‐minute “real” and “sham” tDCS (2 mA) were applied over the DLPFC on two separate days in random order. Each subject received both stimulation conditions with a 1‐week washout period. The anode and cathode electrodes were located over the right and left DLPFC, respectively. Resting state fMRI was acquired before and after real and sham stimulation. Subjective craving was assessed before and after each fMRI scan. The RSNs were identified using seed‐based analysis and were compared using a generalized linear model.

**Results:**

Subjective craving decreased significantly after real tDCS compared to sham stimulation (*p* = .03). Moreover, the analysis shows significant modulation of DMN, ECN, and SN after real tDCS compared to sham stimulation. Additionally, alteration of subjective craving score was correlated with modified activation of the three networks.

**Discussion:**

Given the observed alteration of the targeted functional brain networks in methamphetamine users, new potentials are highlighted for tDCS as a network intervention strategy and rsfMRI as a suitable monitoring method for these interventions.

## 1 INTRODUCTION

Beyond investigation of the contribution of specific brain areas to specific cognitive and behavioral processes, contemporary approaches are extended to address communication between these regions. The human brain can be regarded as a system composed of specialized modules, which interact temporally and/or spatially with each other (Polanía, Nitsche, & Paulus, 2012). Recent studies have revealed the intrinsic organization of the brain into coherent functional networks (Fox et al., 2005; Menon, 2011). The number of theoretical and empirical studies with a network perspective is rapidly increasing; correspondingly, evaluation of large‐scale networks is made possible through the development of new neuroimaging methods. This approach allows us to monitor functional segregation (i.e., regional information processing) as well as integration (i.e., combination of information from different brain regions), which is an important perspective for understanding human brain function as a complex interconnected system. Here, resting state fMRI (rs‐fMRI) is a method to explore spontaneous fluctuations of blood‐oxygen‐level‐dependent (BOLD) signals in different brain regions in the absence of specific cognitive tasks. With rs‐fMRI, it is possible to evaluate alterations of internetwork as well as intranetwork connectivity independent from task‐specific confounding effects of traditional active paradigms (Fedota & Stein, 2015; Lu & Stein, 2014)

Over the last few years, increased interest in understanding how these large‐scale networks connect to cognitive and affective dysfunctions has triggered several studies in psychopathology. A large number of studies revealed disturbances in the functional connectivity of large‐scale brain networks in patients with schizophrenia (Chen et al., 2013; Moran et al., 2013; Palaniyappan, Mallikarjun, Joseph, White, & Liddle, 2011; Zhang et al., 2012), depression (Greicius et al., 2007; Peng et al., 2015; Smith, Allen, Thayer, & Lane, 2015; Zhang et al., 2015), anxiety (Andreescu et al., 2014; Etkin, Prater, Schatzberg, Menon, & Greicius, 2009; Modi, Kumar, Kumar, & Khushu, 2015), and attention‐deficit/hyperactivity disorder (ADHD) (Di Martino et al., 2013; McLeod, Langevin, Goodyear, & Dewey, 2014; Sun et al., 2012). Likewise, alterations of functional connectivity have been investigated in patients with different substance use disorders, such as cocaine, heroin, morphine, nicotine, alcohol, and caffeine (Camchong et al., 2011; Gu et al., 2010; Khalili‐Mahani et al., 2012; Ma et al., 2010; Meunier et al., 2012; Niesters et al., 2012; Sutherland et al., 2013; Tal et al., 2013; Tomasi et al., 2010; Upadhyay et al., 2010; Wong, Olafsson, Tal, & Liu, 2012). Current studies in functional connectivity of addiction are focused on three well‐established intrinsic networks: the default mode network (DMN) which includes the ventromedial prefrontal cortex (VMPFC) and posterior cingulate cortex (PCC), the executive control network (ECN) hooked in the dorsolateral prefrontal cortex (DLPFC) and posterior parietal cortex (PPC), and the salience network (SN) including the anterior insula and anterior cingulate cortex (ACC) (Li et al., 2017; Liang et al., 2015; Qiu et al., 2016; Seeley et al., 2007). Sutherland, McHugh, Pariyadath, and Stein (2012) proposed a heuristic framework to describe the relation between these three networks in the addicted brain. According to this framework, in a nicotine deprivation state, SN would direct attention resources toward internal withdrawal symptoms—thus shifting brain functions toward DMN and away from ECN, whereas under nicotine administration, the SN would direct attentional resources toward external stimuli and executive functions—thus shifting brain activity toward ECN and away from DMN (Lerman et al., 2014; Sutherland et al., 2012). This switch between DMN and ECN is also suggested to be mediated by insula (Sutherland et al., 2012).

In the last decade, transcranial direct current stimulation (tDCS) has been revived as a noninvasive neuromodulation technique that modulates cortical excitability of the human brain. Furthermore, tDCS has been applied as a novel treatment intervention in various neuropsychiatric disorders. Even though, during the first years, investigators were focused on the regional effect of tDCS; recently, interest in evaluation of the effects of tDCS on functional brain networks has increased (Keeser et al., 2011; Peña‐Gómez et al., 2012; Polanía, Paulus, Antal, & Nitsche, 2011).

A recently conducted study resulted in stronger intranetwork functional connectivity for the networks that are known to be anticorrelated with DMN, whereas robustness of the DMN was reduced after tDCS (Peña‐Gómez et al., 2012). Keeser et al. (2011) also described that anodal tDCS over the left DLPFC increased intrinsic functional connectivity within the DMN and the left frontal–parietal network (Keeser et al., 2011); however, they applied a different montage with the anode positioned over the left DLPFC. These results suggest that prefrontal tDCS modulates large‐scale patterns of resting state connectivity in the human brain.

While a couple of studies have reported promising effects of tDCS on drug craving (Boggio et al., 2008, 2009, 2010; Fecteau et al., 2014; Fregni et al., 2008; Klauss et al., 2014; Shahbabaie et al., 2014; da Silva et al., 2013), most of these did not describe the underlying mechanisms of tDCS outcomes due to lack of objective physiological measures such as neuroimaging. According to some addiction‐related Event‐related potential (ERP) studies, an alteration in p300 components after tDCS is evidence for tDCS‐induced increased activity in prefrontal cortex (Conti, Moscon, Fregni, Nitsche, & Nakamura‐Palacios, 2014; Nakamura‐ Palacios et al., 2012). In a more recent ERP study, Nakamura‐Palcious et al. reported increased P3 activation over the ventral medial prefrontal cortex (vmPFC) under drug‐related cues in alcoholics and crack cocaine users during and after the treatment with bilateral tDCS over DLPFC. In crack cocaine users, they also found increased diffusion tensor imaging (DTI) parameters relating to the connection between vmPFC and nucleus accumbens (NAcc); this increase was significantly correlated with craving decrease after repetitive tDCS (Nakamura‐ Palacios et al., 2016). However, these studies merely confirm the role of prefrontal cortex, but not network effects. Hence, the proposed underlying mechanisms involved in the therapeutic effects of tDCS in drug addiction are still subject of speculation (Yavari et al., 2015). In this study, we hypothesized that tDCS over DLPFC, in early abstinent methamphetamine users, would enhance functional connectivity of ECN through increased temporal correlation between DLPFC (the major hub of ECN) and other functionally related regions in this network. As a result, considering the anticorrelated nature of DMN and ECN, we conversely expected decreased functional connectivity in the DMN.

These functional network connectivity alterations could have the potential to explain an important effect of tDCS on drug craving. Drug craving is one of the most important factors in addiction that can lead to drug‐seeking behavior during abstinence including emotional and cognitive aspects along with behavioral and physiological states. Therefore, we further hypothesized that subjective craving might be associated with the respective functional connectivity alterations.

## 2 MATERIALS AND METHODS

### 2.1 Subjects

This study was part of a larger fMRI study investigating the effects of tDCS on neural substrates in patients with methamphetamine use disorders (MUD) with at least 1‐week abstinence confirmed by negative urine analysis. All recruited subjects were under a course of abstinence‐based therapy in the Omid Javid Residential Center, Tehran Welfare Organization. Fifteen right‐handed males (age; mean ± *SE*: 31.33 ± 1.40 years) who met our inclusion/exclusion criteria entered this study (see Table 1 for demographic information). After signing a written informed consent, the semistructured interview was conducted by a licensed psychiatrist to identify patients who had a history of minimum 6 months of MUD and no current psychiatric disorders based on DSM‐5 axis I, except for substance use disorders. Moreover, these subjects had no history of either major neurological diseases such as traumatic brain injury, stroke, seizure, and epilepsy or metal brain implants. The study was designed based on the Declaration of Helsinki and was approved by the independent ethical committee of Tehran University of Medical Sciences.

**Table 1:** Demographic characteristics

	Descriptive statistics (Mean ± *SE*)
Gender (male)	15/15
Age	31.33 ± 1.40
Education (years)	11.73 ± 0.64
Duration of MUD^a^(days)	13.31 ± 1.19
Duration of SUDs^b^ (years)	3.87 ± 0.59
Age at the onset of MUD	25.26 ± 1.49
Age at the onset of SUDs	17.80 ± 1.33
Consumption in last month of abuse (days)	16.20 ± 2.74

a Methamphetamine use disorder.

b Substance use disorder.

### 2.2 Procedure

**Figure 1:**
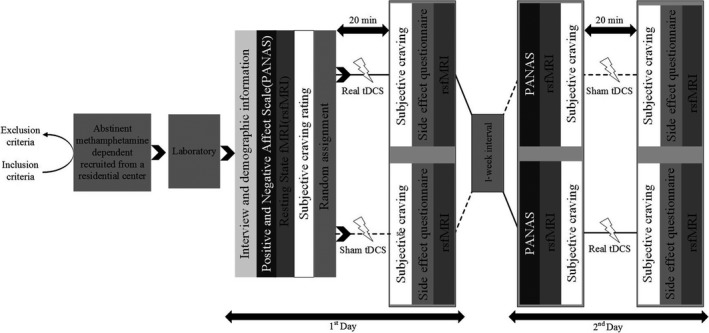
Experimental procedure. At the first session of the experiment, subjects were interviewed and their affective state was evaluated by the Positive and Negative Affect Scale (PANAS). Before and after each transcranial direct current stimulation (tDCS) session, an resting state fMRI (rs‐fMRI) scan was conducted and subjective craving was recorded, respectively. The experiment was conducted in a crossover design, and each subject was randomly assigned to tDCS conditions

The experiment was a randomized double‐blinded sham‐controlled crossover study. Initially, each subject was seated on a comfortable chair and was asked to complete the Persian version of the Positive and Negative Affect Scale (PANAS) in order to control for his affective status before each session. Each subject underwent a counterbalanced study design with two stimulation conditions (real and sham) at two separate days with a 1‐week washout period. Resting state fMRI was acquired before and after each stimulation session. Also, subjects rated their immediate methamphetamine craving before and after each stimulation, with a score range from 0 to 100, where 0 and 100 indicated “no craving” and “extreme craving,” respectively. We assessed possible side effects using a tDCS side effect checklist at the end of each session (Figure 1).

### 2.3 Transcranial direct current stimulation

Direct current was applied via a pair of electrodes (5 cm × 7 cm). These electrodes were made of highly conductive carbon rubber and covered with saline‐soaked sponges. The electrodes were connected to a battery‐driven constant current stimulator (ActivaDose® II Iontophoresis Delivery Unit, USA). In this study, the anode and cathode electrodes were placed over the right and left DLPFC, respectively. Right and left DLPFC are consistent with F4 and F3 based on the international 10–20 system of EEG electrode placement. A couple of studies reported promising findings in reducing subjective craving with this electrode montage (Boggio et al., 2008, 2010; Fecteau et al., 2014; Nakamura‐ Palacios et al., 2016). For the real DC stimulation, 2 mA current intensity was applied for 20 min including a 30‐s ramp. The sham protocol was identical to the real tDCS condition in every aspect except that the tDCS device remained on the subject's scalp for 19.5 min with no stimulation, thus reducing the total stimulation time to 30 s, 15 s of ramp‐up, and 15 s of ramp‐down. Several studies have suggested that the sham method is a reliable control, and the subjects could not discriminate sham from the real stimulation (Brunoni et al., 2012; Gandiga, Hummel, & Cohen, 2006; Kekic et al., 2014, 2017). Furthermore, the stimulation conditions were administered by an expert tDCS technician who was not involved in any measurement processes. Therefore, neither the researchers nor the subjects were informed about the tDCS conditions.

### 2.4 MRI acquisition

Structural and functional brain scans of all subjects were acquired by MRI (Siemens TIM Trio 3 Tesla) at the Neuroimaging and Analysis Group of Tehran University of Medical Sciences. During resting state data acquisition (7.33 min), individuals were asked to fix their eyes on the screen and not to think about anything. The images were acquired by functional imaging EPI sequences with the following parameters: number of volumes = 200; number of slices = 40; repetition time (TR) = 2,200 ms; echo time (TE) = 30 ms; percentage phase field of view (FOV) = 100; matrix size = 64 × 64; slice thickness = 3 mm; interstice gap = 0 mm; flip angle = 90°; spatial resolution = 3 × 3 mm; FOV = 192 × 192 mm^2^.

The parameters for structural images were as follow: TR = 1,800 ms; TE = 3.4 ms; FOV = 256 × 256 mm^2^; flip angle = 7°; slice thickness = 1 mm, number of slices = 176.

### 2.5 MRI processing

FMRI data were preprocessed by the FEAT‐FSL toolbox from the FMRIB software library v5.0.7 (FMRIB software library http://www.fmrib.ox.ac.uk/fsl;RRID:SCR_002823). Preprocessing steps included the following: (1) Brain extraction using BET, (2) Motion correction using MCFLIRT, (3) Interleaved slice‐timing correction, (4) Spatial smoothing using Full width at half maximum (FWHM) = 5 mm, (5) Intensity normalization, and (6) Temporal high‐pass filtering. Two subjects were discarded from further analysis due to severe head motion.

MRI resting state functional connectivity was analyzed by the Analysis of Functional NeuroImages software (AFNI) version 31.12.15 (RRID:SCR_002823). The rsfMRI data were further preprocessed using AFNI. The *3dWarp* structure was used in order to transform an oblique to a cardinal dataset. We used *@auto_tlrc* to spatially transform the images from their original native space to the Talairach space. Seeds were specified based on our theoretical hypotheses from previous publications (Andrews‐Hanna, Reidler, Sepulcre, Poulin, & Buckner, 2010; Seeley et al., 2007), and a cross‐correlation analysis was conducted to extract large‐scale resting state networks (RSNs). Seeds included DMN, bilateral PCC (Talairach coordinates: −1, −50, 26); ECN, right DLPFC (Talairach coordinates: 44, 36, 20); and SN, right orbital anterior insula (Talairach coordinates: 38, 26, −10). These regions of interest (ROIs) were spherical seeds with 6‐mm radius. In the first processing step, our desired ROIs were created by the *3dUndump* function. Then, in order to obtain correlation maps between the ROIs and the whole‐brain time series, we applied the *3dfim+* function. The Pearson's correlation coefficient was converted to Fisher's Z transformation using *3dcalc* to make inferences.

### 2.6 Statistical analysis

Effects of demographic and psychological characteristics were analyzed using SPSS version 21.0 (SPSS, Inc., Chicago, IL, USA; RRID:SCR_002865). In order to identify significant clusters for the desired networks, we applied one‐sample *t* tests for each condition (before and after real tDCS, before and after sham tDCS) and Monte Carlo simulated correction was adopted (α = .05 voxel‐wise *p* < .01, cluster size >726 mm^3^).

To identify the effect of tDCS on the three RSNs, the (postreal >prereal) > (postsham >presham) contrast was examined using two‐sample paired *t* tests. To assure that the observed effect is not affected by baseline differences, a paired *t* test was performed to compare pre‐tDCS images of the two conditions. The results were explored for significance by the Monte Carlo simulation algorithm (α = .05, voxel‐wise *p* < .05, cluster size >4,089 mm^3^).

To explore the changes of subjective self‐reported craving in association with differential connectivity of the respective brain networks, a multiple linear regression approach was employed as follows:

$$\begin{array}{cc} y_{i}= & \beta_{0}+\beta_{1}\times \text{VAS}+\beta_{2}\times \text{Dur. of Abstinence}+\\ & \beta_{3}\times \text{Dur. of Substance Use Disorder} \end{array}$$

where *y*_*i*_ is the voxel‐wise value of changes in each network across subjects, β_0_ is the intercept of straight‐line fitting in the model. β_1_, β_2_, β_3_ are the effects of changes in subjective craving, duration of abstinence, and duration of substance use disorder on functional connectivity of *i*th voxel in each network contrasting real versus sham tDCS. Effects of β_2_ and β_3_ were ignored as covariates of no interest in the linear regression model. The voxel‐wise multiple linear regression map was corrected by the Monte Carlo simulation test (α = .05, voxel‐wise *p* < .05, cluster size >4,089 mm^3^) in order to demonstrate the significant neural correlates of subjective craving, after controlling for duration of abstinence and duration of substance use disorder in the mentioned networks.

## 3 RESULTS

### 3.1 Demographic and psychological characteristics

According to the PANAS questionnaire, there was no significant difference, neither in positive nor in negative affect, between the two sessions, as shown by the Wilcoxon test (*p* > .05). Thus, patients entered the experiment without significant difference in their affective states on both days. Dominant pattern of methamphetamine use was smoking in all subjects (for more demographic characteristics, see Table 1). Subjective craving after real and sham stimulation examined by the Wilcoxon test showed a significant reduction in immediate craving after real compared to sham tDCS (mean score change in real session = −15.42 ± 5.42 *SE*, mean score change in sham session = −1 ± 2.63 *SE*;* p* = .03). Moreover, there was no significant difference in baseline craving measurement between sham and real conditions (mean ± *SE*: before real‐tDCS = 17.33 ± 3.88; before sham‐tDCS = 22.30 ± 5.70; *p* = .43). TDCS was well tolerated by all participants without any major complications, and the adverse effects did not differ between real and sham tDCS according to chi‐squared test (*p* > .05).

### 3.2 Network results

#### 3.2.1 Default mode network activities in baseline imaging

**Figure 2:**
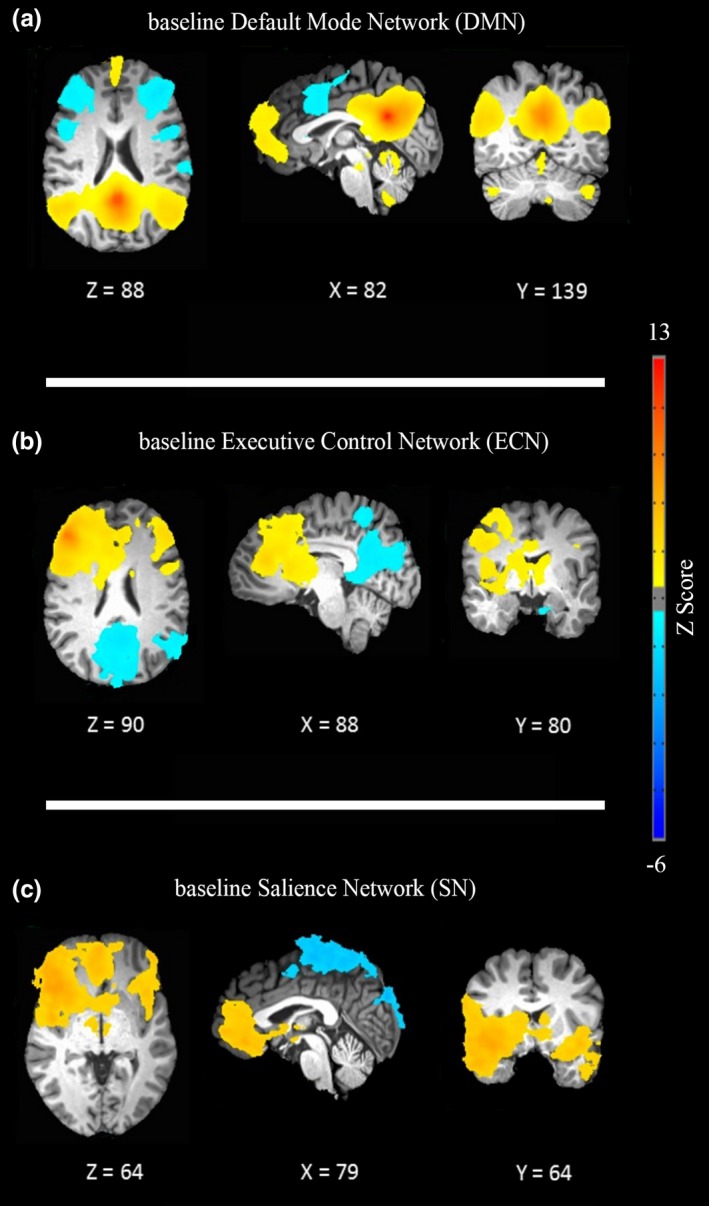
Large‐scale brain networks before stimulation (baseline). Networks including (a) default mode network (DMN), (b) executive control network (ECN), and (c) salience network (SN) were extracted by one‐sample *t* tests after multiple comparison correction (α = .05 voxel‐wise *p* < .01, cluster size >726 mm^3^). The color bar shows *Z* scores. Overlaid networks are illustrated in a blue–red spectrum where blue and red continua indicate negative and positive connectivity, respectively. Network maps are displayed following radiological (left = right) convention based on the Talairach coordination system

Baseline DMN connectivity group analysis revealed positive correlations between the following regions with the DMN seed, based on the Talairach Daemon (TD) database: precuneus (Brodmann area [BA]: 31) PCC (BA: 23/30/31) superior/middle temporal gyrus bilaterally (BA: 22 /21), medial frontal gyrus bilaterally (BA: 10), ACC (BA: 32). Anticorrelated regions were as follows: superior/middle /inferior frontal gyrus bilaterally (BA: 9/46/9) and insula (BA: 13). The activation pattern of baseline DMN is illustrated in Figure 2a.

#### 3.2.2 Default mode network after real versus sham tDCS

**Figure 3:**
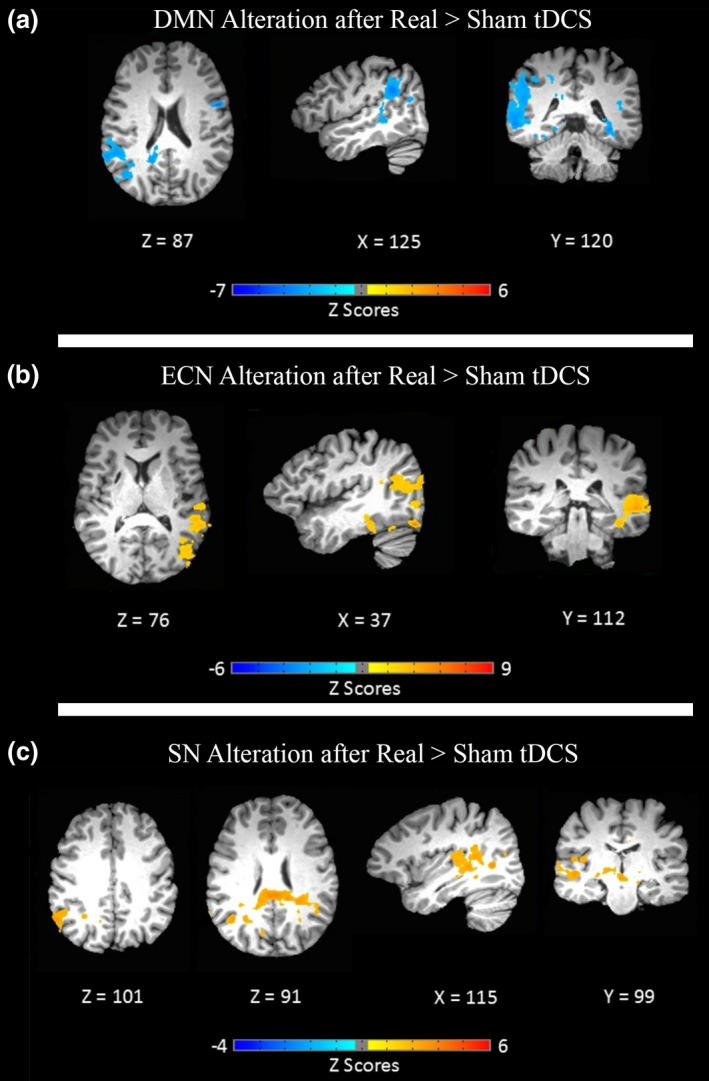
Effects of transcranial direct current stimulation (tDCS) on large‐scale brain networks. Connectivity alterations of the large‐scale brain networks were identified by paired *t* tests after multiple comparison correction (α = .05, voxel‐wise *p* < .05, cluster size >4,089 mm^3^). (a) default mode network (DMN), (b) executive control network (ECN), and (c) salience network (SN) were modulated after real versus sham tDCS

The group analysis shows significantly decreased connectivity for the contrast ([postreal > prereal] > [postsham > presham]), comparing postreal vs. sham tDCS. This decrease was observed in two clusters as follows: Cluster 1: right middle temporal gyrus (BA: 39), right superior temporal gyrus (BA: 21/41/42), right supramarginal gyrus (BA: 40), right inferior parietal lobule, right precuneus (BA: 31), and right PCC (BA: 23). Cluster 2: left superior temporal gyrus (BA: 22), left precentral gyrus (BA: 6/44), left middle temporal gyrus (BA: 21), and left inferior frontal gyrus (BA: 44). It is worth mentioning that there were no differences between pre‐tDCS DMN in two conditions. The respective DMN modulation pattern is shown in Figure 3a and Table 2.

**Table 2:** Effects of transcranial direct current stimulation on resting state network connectivity ([Postactive > baseline1] > [Postsham > baseline2])

Networks	Cluster	Brain area	Brodmann's area	Cluster's size	Talairach coordinates (LPI)			Z score (Max)
					*X*	*Y*	*Z*	
Default mode network	1	R middle temporal gyrus	39	19,819	42	−64	22	−3.06
		R superior temporal gyrus	21/41,42		50	−36	12	−2.95
		R supramarginal gyrus	40		48	−46	31	−4.28
		R inferior parietal lobule			48	−47	25	−3.52
		R precuneus	31		19	−52	34	−2.72
		R posterior cingulate cortex (PCC)	23		15	−51	23	−3.55
	2	L superior temporal gyrus	22	6,119	−45	−42	16	−2.72
		L precentral gyrus	6/44		−49	0	16	−4.01
		L middle temporal gyrus	21		−53	−17	−7	−3.46
		L inferior frontal gyrus	44		−48	0	16	−4.77
Executive control network	1	L middle temporal gyrus	21	28,165	−56	−32	0	4.15
		L superior temporal gyrus	22		−60	−53	15	4.37
	2	R supramarginal gyrus	40	10,068	60	−50	35	4.08
		R inferior parietal lobule			59	−57	44	7.83
Salience network	1	R lingual gyrus	18	58,703	19	−59	4	4.52
		R middle temporal gyrus	21		69	−39	3	3.83
		R superior temporal gyrus	22		72	−38	4	4.19
		R PCC	23		2	−34	24	2.97

#### 3.2.3 Executive control network activities in baseline imaging

Positive correlations were observed for baseline ECN connectivity in the following regions: superior/middle/inferior frontal gyrus bilaterally (BA: 9/10/46/9), right anterior cingulate (BA: 32), and insula bilaterally (BA: 13). Furthermore, group analysis of baseline ECN showed negative correlation in the following regions: left superior/middle temporal gyrus (BA: 22/39/19), precuneus (BA: 30), cuneus (BA: 18/23/31), and PCC (BA: 31/23). Figure 2b illustrates the baseline ECN activation pattern.

#### 3.2.4 Executive control network after real versus sham tDCS

Regions with increased activation in ECN ([postreal > prereal] > [postsham > presham]) are as follows: Cluster 1: left middle temporal gyrus (BA: 21), and left superior temporal gyrus (BA: 22). Cluster 2: right supramarginal gyrus (BA: 40), and right inferior parietal lobule (Figure 3b and Table 2). Additionally, paired *t* test showed no differences between presham and prereal ECN.

#### 3.2.5 Salience network activities in baseline imaging

Positive correlations were observed between regions of baseline SN connectivity including bilateral middle/inferior frontal gyrus (BA: 11/47), superior temporal gyrus (BA: 38), and insula (BA: 13). Regions with negative correlation for baseline SN include the following: left inferior parietal lobule (BA: 7/40), left precuneus (BA: 31), left precentral gyrus (BA: 4), and left postcentral gyrus (BA: 1/2/3). The SN activation pattern is exemplified in Figure 2c.

#### 3.2.6 Salience network after real versus sham tDCS

Tuned up SN regions in ([postreal > prereal] > [postsham > presham]) contain the following: right lingual gyrus (BA: 18), right superior/middle temporal gyrus (BA: 22/21), and right PCC (BA: 23). SN connectivity alterations are shown in Figure 3c and Table 2. According to paired *t* test results, prereal SN was not statistically different from presham SN.

### 3.3 Network activities associated with subjective craving changes

#### 3.3.1 Default mode network

**Figure 4:**
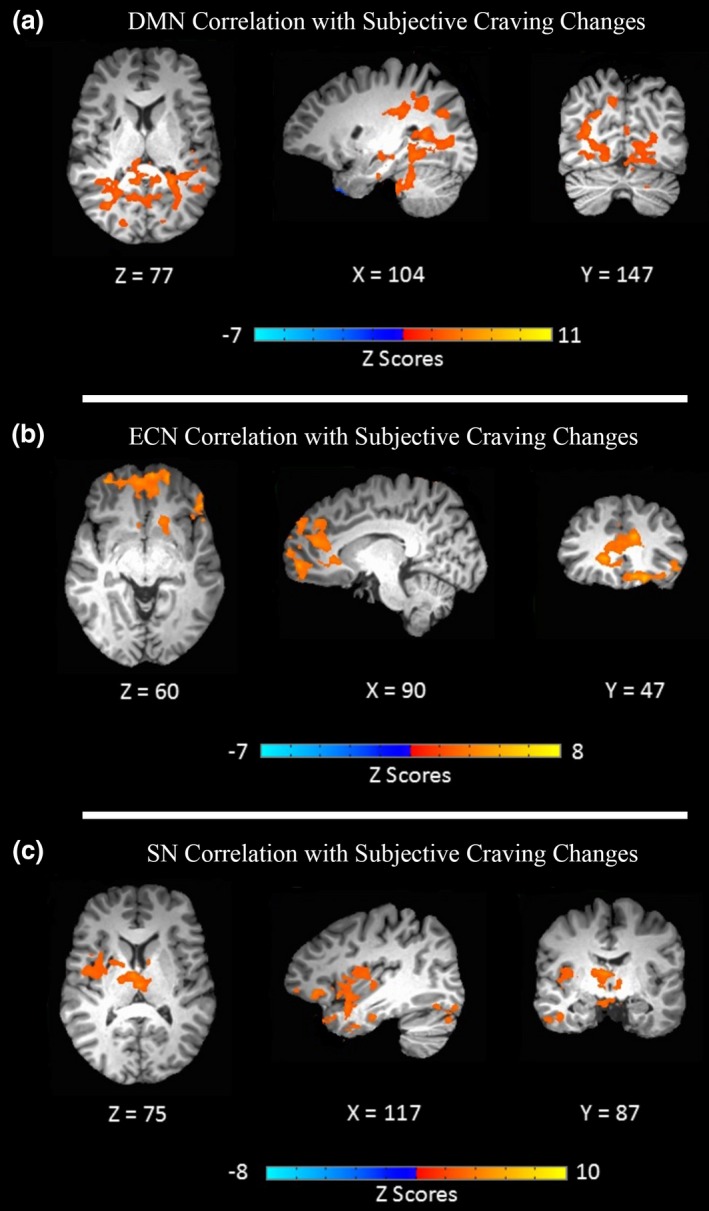
Association of network modulation and subjective self‐reported craving. Significant neural correlates of subjective craving were extracted by multiple linear regression analysis after controlling for duration of substance use disorders and duration of abstinence (corrected by Monte Carlo simulation α = .05, voxel‐wise *p* < .05, cluster size >4,089 mm^3^). Connectivity alterations ([postreal > prereal] > [postsham > presham]) which were associated with subjective craving changes (post–pre) are illustrated for (a) default mode network (DMN), (b) executive control network (ECN), and (c) salience network (SN)

Decreased subjective craving was positively correlated with less connectivity in a cluster including the right/left lingual gyrus (BA: 18/19), parahippocampal gyrus bilaterally (BA: 30), right precuneus (BA: 7), right posterior cingulate gyrus (BA: 29), and left middle temporal gyrus (BA: 22) (Figure 4a, Table 3).

**Table 3:** Correlation of subjective craving changes with resting state network connectivity alterations ([Postactive > baseline1] > [Postsham > baseline2])

Networks	Cluster	Brain area	Brodmann's area	Cluster's size	Talairach coordinates (LPI)			*Z* score (Max)
					*X*	*Y*	*Z*	
Default mode network	1	R lingual gyrus	19	109,174	19	−73	1	3.82
		L lingual gyrus	18/19		−14	−65	−1	3.1
		R precuneus	7		8	−60	39	3.48
		L parahippocampal gyrus	30		−25	−49	6	6.66
		L middle temporal gyrus	22		−54	−36	1	3 .94
		R parahippocampal gyrus	30		32	−46	6	4.89
		R posterior cingulate gyrus	29		7	−46	18	5.31
Executive control network	1	R medial frontal gyrus	10	32,000	17	42	20	4.18
		L medial frontal gyrus	10		−6	62	−5	4.82
		R superior frontal gyrus	10		16	70	12	5.08
		R anterior cingulate gyrus	10		15	32	4	5.09
		L superior frontal gyrus	10		−19	58	−7	4.92
		L anterior cingulate gyrus	32		−8	34	23	4.84
		L inferior frontal gyrus	47		−46	33	−4	3.63
		L middle frontal gyrus	11		−20	32	−13	5.19
		R middle frontal gyrus	11		27	43	−12	7.68
	2	L precuneus	7	15,517	−3	−60	48	5.13
		R precuneus	7		1	−53	44	3.14
Salience network	1	R insula	13	27,926	40	16	2	3.74
		R inferior frontal gyrus	47 /13		43	19	1	3.2
		R middle frontal gyrus	11		39	32	−6	4.48
		L Thalamus			−1	−14	10	4.42

#### 3.3.2 Executive control network

Reduction in craving was correlated with increased functional connectivity in some parts of ECN such as cluster 1: medial frontal gyrus bilaterally (BA: 10), left/right superior frontal gyrus (BA: 10), and left /right ACC (BA: 10), inferior frontal gyrus (BA: 47), and bilateral middle frontal gyrus (BA: 11). In addition, cluster 2: left/right precuneus were correlated with subjective craving (Figure 4b, Table 3).

#### 3.3.3 Salience network

Higher functional connectivity in right insula (BA: 13), inferior frontal (BA: 47/13), right middle frontal gyrus (BA: 11), and left thalamus was associated with reduced subjective craving (Figure 4c, Table 3).

## 4 DISCUSSION

Analyses of rs‐fMRI data following brain stimulation showed that single‐session bilateral tDCS over the DLPFC alters functional connectivity of the relevant large‐scale networks in early abstinent methamphetamine users. More specifically, intranetwork functional connectivity of DMN decreased while both ECN and SN increased intranetwork functional connectivity. Additionally, these alterations were associated with reduction in subjective self‐reported craving. Finally, there was no significant change in participant's affective states after tDCS as measured by PANAS. Hence, it is safe to assume that the results are not attributed to mood changes.

### 4.1 Effects of tDCS on RSNs

In line with our hypotheses, the results of the current study demonstrate that application of bilateral tDCS with the anodal electrode over the right DLPFC decreases synchronized temporal activity within the DMN. This diminished functional connectivity was largest for posterior parts of DMN including the following: right middle temporal gyrus (BA: 39), right precuneus, and right PCC. DMN is a well‐established intrinsic large‐scale network that contributes to self‐referential monitoring and is deactivated when goal‐directed and cognitive tasks are performed (Boettiger, Chanon, & Kelm, 2013; Gusnard, Akbudak, Shulman, & Raichle, 2001; Hamilton et al., 2011). Abnormalities of DMN have also been reported in substance use disorders (Ding & Lee, 2013; Li et al., 2013; Ma et al., 2011). Sutherland et al. (2012) developed a network model for addiction which suggests that for withdrawal symptoms, characterized by affective and /or motivational disturbances (including craving) in abstinent states, the insula redirects attentional sources to endogenous stimuli in order to eliminate these problems, and therefore functional connectivity of DMN increases. According to this model, we speculate that anodal tDCS over the right DLPFC decreases functional connectivity of DMN which is followed by redirection of attentional resources to external stimuli, hence reducing withdrawal symptoms as well as drug craving. Although there are a few tDCS studies that address DMN modulation (Kajimura, Kochiyama, Nakai, Abe, & Nomura, 2016; Keeser et al., 2011; Peña‐Gómez et al., 2012), our findings are directly consistent with Peña‐Gómez et al. (2012) that reported decreased functional connectivity in DMN after anodal stimulation of the right DLPFC. Keeser et al. (2011) showed increased DMN connectivity after prefrontal tDCS; however, they applied a different montage with the anode positioned over the left DLPFC.

Analysis of the ECN revealed that intranetwork functional connectivity increased after bilateral tDCS with anodal stimulation of the right DLPFC. This increase was significant in the posterior part of the right inferior parietal lobule. Resting state ECN is a task‐positive network, which despite its importance, has been less well studied in comparison with DMN. ECN is implicated in executive functions and goal‐directed cognition (Boettiger et al., 2013; Spreng, Stevens, Chamberlain, Gilmore, & Schacter, 2010; Wu et al., 2014). There are few evidences of functional impairments in the ECN and executive function in addiction (Carmichael & Lockhart, 2012; Dong, Lin, & Potenza, 2015; Krmpotich et al., 2013; Weiland et al., 2014), which are worsened during early abstinence (Copersino et al., 2004; Jacobsen, Pugh, Constable, Westerveld, & Mencl, 2007). Moreover, based on the network model suggested by Sutherland et al., (2012), increased functional connectivity of the ECN could reduce withdrawal symptoms while enhancing the process of external and task‐related activity. Furthermore, a recent study showed that anodal tDCS over the DLPFC increases intranetwork coactivation of the ECN (Cavaliere et al., 2016). We thus suggest that anodal stimulation of the right DLPFC improved intranetwork functional connectivity of ECN, and consequently, sources of attention are switched to external stimuli. That being said, the present work can also explain why most tDCS studies which target DLPFC reported efficient results in a wide range of cognitive functions.

In addition, our findings also demonstrate increased connectivity within the SN after tDCS over the DLPFC. It is suggested that the SN monitors relevant stimuli indiscriminately, dynamically switches the attentional resources to internal or external stimuli and has a causal role in DMN–ECN oscillations (Menon & Uddin, 2010; Seeley et al., 2007; Sridharan, Levitin, & Menon, 2008). Given the increased functional connectivity of both ECN and SN after tDCS, we argue that tDCS improves intranetwork connectivity of SN and hence turns the attentional resources toward external stimuli, which results in increased functional connectivity of the ECN. Following increased connectivity within the ECN, we expect reduced craving due to redirection of attentional resources toward external (nonself‐referential) stimuli and away from drug‐related stimuli through SN.

### 4.2 Limitations

One important limitation of the current study is the small sample size, which reduces statistical power. Nevertheless, we applied a crossover design, which optimizes statistical power despite small sample size and further eliminates the effects of individual differences. The initial alpha level is also chosen according to the exploratory nature of the current study. Replicating this study with larger sample sizes and a more stringent alpha level is proposed for future confirmatory studies. Another limitation of our study is related to the bipolar electrode montage, which does not allow to decide which electrodes were relevant for the effects. The third limitation is that the current study only addressed three important addiction‐related RSNs including DMN, ECN, and SN which have consistently been reported, while other large‐scale networks such as memory and attention networks have also been of interest in the literature (Kelly et al., 2011; Zhai et al., 2014). So, future studies considering additional networks are recommended for expanding knowledge and generating new insight into the subject matter. Finally, comparing the same outcomes in a healthy control group could have been beneficial; however, drug craving is assumed to be a dependent variable in this study and we assumed that it may not make sense to study craving in healthy controls. To overcome this limitation, it might be possible for future studies to examine methamphetamine users and healthy subjects in the same context.

## 5 CONCLUSION

To our best knowledge, this is the first study that applies tDCS to modulate large‐scale brain networks in a specific drug use disorder. The results of this study show that not only tDCS modulates functional connectivity in large‐scale human brain networks, but also that these changes are correlated with the reduction in drug craving. Moreover, our findings support the widespread theoretical framework suggested by Sutherland et al. (2012). However, the current study is a single‐session tDCS and may influence the brain networks in a different way from repetitive tDCS. Therefore, more studies and multisession clinical trials are needed to ascertain the use of tDCS as a therapeutic technique to improve dysfunctions of intrinsic large‐scale networks in chronic drug users and by this reduce clinical symptoms.

## DISCLOSURE

The authors declare no potential conflicts of interests.
